# The assessment of proinflammatory cytokines in the patients with the history of cerebral venous sinus thrombosis

**Published:** 2016-04-03

**Authors:** Farnaz Akbari, Askar Ghorbani, Farzad Fatehi

**Affiliations:** ^1^ Iranian Center of Neurological Research, Shariati Hospital, Tehran University of Medical ‎Sciences, Tehran, Iran

**Keywords:** Cerebral Thrombosis, Venous Thromboembolism, Interleukins, Cytokines, Erythrocyte Sedimentation Rate

## Abstract

**Background:** Evidence is accumulating that venous thromboembolism is not limited to coagulation system and immune system seems to be involved in formation and resolution of thrombus. Some studies have demonstrated the role of inflammatory factors in deep venous thrombosis (DVT) of limbs; however, there has not been such study in the patients with cerebral venous sinus thrombosis (CVST). The purpose of this study was to evaluate inflammatory cytokines including interleukin-6 (IL-6), IL-8, IL-10, and tumor necrosis factor-alpha (TNF-α) in the patients with the history of CVST.

**Methods:** In a cross-sectional study, 20 patients with the first episode of CVST and 20 age- and sex-matched healthy controls were included. The patients were seen only after anticoagulant treatment had been discontinued for at least 3 months. IL-6, IL-8, IL-10, TNF-α levels, and erythrocyte sedimentation rate (ESR) were measured in two groups.

**Results:** The median age of patients was 37.0 [interquartile range (IQR) = 31.75-42.75] and in control group was 42.0 (IQR = 38.0-40.6) (P = 0.18). In patients group, 14 (70%) were females and in control group, also, 14 (70%) subjects were female (P = 0.01). It is significant that the level of IL-6 was significantly higher in the control group [patients: median: 9.75, IQR: 8.98-10.65; controls: median: 11.45, IQR: 10.28-13.10; P = 0.01]; however, the ESR level was higher in the patients. On the subject of IL-8, IL-10, and TNF-α, no significant difference was detected.

**Conclusion:** We did not find higher concentrations of inflammatory ILs in the patients with the history of CVST that is contradictory with some findings in venous thrombosis of the extremities; however, the studies with larger sample size may be required.

## Introduction

Classic risk factors for venous thrombosis are divided into two main groups of acquired factors such as immobilization, surgery and cancers, and genetic risk factors, like activated protein C resistance, and deficiencies of protein C or S and antithrombin.^1^ Evidence is accumulating that venous thromboembolism is not limited to coagulation system and immune system seems to be involved in formation and resolution of thrombus.^2^^,^^3^

Cytokines are different groups of soluble short acting proteins, glycoproteins, and peptides produced by numerous immune cells and vascular cells, and act in picomolar to nanomolar concentrations to trigger specific receptors and modulate the functions of many cells and tissues.^4^ Interleukins (ILs) are cytokines synthetized by one leukocyte and acting on other leukocytes. Anti-inflammatory cytokines are involved in the down-regulation of inflammatory reactions such as IL-10 and some others such as tumor necrosis factor-alpha (TNF-α), IL-6 provoke stimulation of acute-phase reactants or chemoattractant such as IL-8.^4^

There is evidence that elevated levels of ILs could be associated with venous thrombosis.^5^^-^^7^ Elevated plasma levels of IL-8 were previously shown to be associated with recurrent venous thrombosis.^8^ In addition, in particular, IL-6, IL-8, and TNF-α play an important role in the process of inflammation and thrombosis formation. IL-6 provokes a prothrombotic effect by increasing expression of tissue factor, fibrinogen, factor VIII and Von Willebrand factor (VWF), activation of endothelial cells and accumulating platelet creation; in addition, it decreases the levels of inhibitors of hemostasis such as anti-thrombin and protein S.^9^

Cerebral venous sinus thrombosis (CVST) is an uncommon cerebrovascular disease representing approximately 1% of all strokes, marked by clotting of blood in cerebral venous or dural sinuses, and in rare cases, cortical veins.^10^ A great many of risk factors have been previously described for the CVST patients.^10^ Previously, local and generalized infections had an important role in the pathogenesis of CVST; but the studies in recent years have shown that in addition to acquired risk factors such as oral contraceptive pills, inherited blood coagulation disorders play an important role in the development of CVST.^10^

Some studies have demonstrated the role of inflammatory factors in deep venous thrombosis (DVT) of limbs; however, there has not been such study in CVST patients. We tested the hypothesis that a chronic inflammatory state following a proinflammatory stimulus, regardless of origin, could precede future thrombotic events. The purpose of this study was to evaluate proinflammatory markers including IL-6, IL-8, IL-10, and TNF-α in the patients with the history of CVST in comparison with healthy individuals.

## Materials and Methods

This was a cross-sectional study conducted between January 2013 and June 2015. A total of 20 patients with a first episode of objectively demonstrated CVST and 20 age- and sex-matched healthy controls were included. The patients had been previously hospitalized in Shariati Hospital, affiliated to Tehran University of Medical Sciences, Tehran, Iran.

The patients were seen only after anticoagulant treatment had been discontinued for at least 3 months. This was always at least 6 months after the event because oral anticoagulant treatment was routinely given for 3-9 months in unprovoked CVST patients. The healthy control subjects were acquaintances of patients or partners of other patients and were selected according to the following criteria: same sex, same age (± 5 years), no biological relationship, no history of venous thromboembolism, no use of warfarin-derivatives for at least 3 months, and no known malignancies.

Inclusion criteria for the patients were definite diagnosis of CVST according to history, clinical exam, and brain magnetic resonance imaging (MRI) and magnetic resonance volumetry. The clinical records and objective documentation of CVST were reviewed by a neurologist to confirm the diagnosis. The age was between 18 and 65 years.

The exclusion criteria were the CVST patients with the history of surgical intervention, stupor or coma during hospitalization, history of ischemic heart disease, intermittent claudication and arterial stroke or transient ischemic attacks, history of acute or chronic renal failures, the previous diagnosis of autoimmune disorders previous to CVST or diagnosed after CVST, secondary thromboses as a result of malignancy, pregnancy, infectious disorders, and thrombophilia, recent immobility, history of diabetes mellitus (DM), smoking and hypertension. Of utmost importance, the patients who required life-long anticoagulation (due to hereditary thrombophilia, recurrent thrombosis, and unresolvable thrombophilic risk factor) were not enrolled in the study.

We drew 10 ml blood and transferred to the laboratory during 1 hour. Enzyme-linked immunosorbent assay (ELISA) kits for IL-6, IL-8, IL-10 and TNF-α were used from Diaclone Company, France. Whole-blood samples were collected in the early morning after overnight fasting. The samples were stored for 15 hours at room temperature and then centrifuged at 1000 × g for 20 minutes. The supernatant was then separated and stored at −70 °C until testing. Repeated freezing and thawing were avoided for all samples. The procedures for the measurement of IL-6, IL-8, IL-10, TNF-α level followed the instructions provided by the kit manufacturers. Erythrocyte sedimentation rate (ESR) was measured with Westergren method.

All patients or their caregivers/relatives provided signed consent to participate in this study. This study was approved by Ethical committee of Tehran University of Medical Sciences.

Data analysis was performed using RStudio (version 3.2.2). Since the data did not follow normal distribution, we used the non-parametric Mann–Whitney test for analysis (comparison of the factors between patients and controls). The significance level below 0.05 was regarded significant. The data are presented as median [interquartile range (IQR: 25-75^th^ percentiles) range].

## Results

The median age of patients was 37.0 years (IQR = 31.8-42.8) and in the control group was 42.0 years (IQR = 38.0-40.6) (P = 0.18). In patients group, 14 (70%) were females and in the control group, 14 (70%) subjects were female (P = 0.01). In CVST group, 5 women consumed oral contraceptive pills before the episode. Patients were included in the study at different time intervals after the thrombotic episode [median 10.5 months (IQR = 9.0-12.0)]. All patients had received during acute-phase and the median duration of warfarin consumption was 6 months (IQR = 5.0-6.3). 

**Figure 1:**
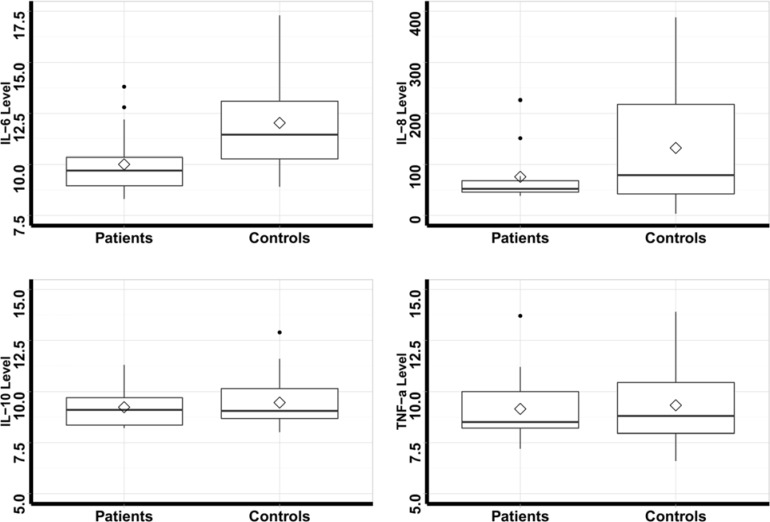
The comparison of measured factors between two groups of patients and controls (the levels are in pg/ml)

The comparison of blood levels of measured factors (IL-6, IL-8, IL-10, TNF-α, and ESR) are shown in table 1 and figure 1. It is significant that the level of IL-6 was significantly higher in the control group (Table 1, Figure 1); however, the ESR level was higher in the patients. On the subject of IL-8, IL-10, and TNF-α, no significant difference was detected.

**Table 1:** The measured factors between patients and controls

**Factors**	**Patients (n = 20)**	**Controls (n = 20)**	**Mann-Whitney U**	**P**
	**Median IQR (25-75** ^th^ ** percentiles)** **‎**	**Median IQR (25-75** ^th^ ** percentiles)** **‎**		
IL-6 (pg/ml)	9.75 (8.98-10.65)	11.45 (10.28-13.10)	105.00	0.01
IL-8 (pg/ml)	53.00 (46.25-72.50)	158.50 (45.0-301.8)	148.50	0.17
IL-10 (pg/ml)	9.10 (8.36-9.83)	9.05 (8.38-9.83)	196.50	0.93
TNF-α (pg/ml)	8.65 (8.20-10.15)	8.80 (7.98-10.68)	191.00	0.82
ESR (mm/hour‎)	13.50 (9.75-24.25)	9.50 (8.00-11.25)	114.00	0.02

ESR: Erythrocyte sedimentation rate; TNF-α: Tumor necrosis factor-alpha; IL: Interleukin; IQR: Interquartile range

## Discussion

According to our results, we could not confirm the hypothesis that pro-inflammatory cytokines are higher in the patients with the history of CVST; however, there have been some studies in favor of this hypothesis in DVT of extremities.

In van Aken et al. study,^8^ the patients with the first event of objectively demonstrated DVT were involved in whom anticoagulant treatment had been discontinued for at least 3 months (at least more than 6 months after the event). The study demonstrated that higher concentrations of IL-8 were related to venous thrombosis. Plasma concentrations of IL-8 (above the 90^th^ percentile) lead to around two-fold increased risk of venous thrombosis, and this association was most noticeable between 40 and 51 years. However, in our study, we did not find such difference between CVST patients and controls.

It is significant that in a population of cancer patients with abdominal malignancies, the C-reactive protein, IL-6, nuclear factor-κB, and E-Sel levels in patients with DVT were meaningfully higher than the control group. The IL-10 level was higher in patients with DVT than in controls.^11^ Another study demonstrated that IL-10 -1082A/G polymorphism is associated with increased risk of DVT.^12^

In a case-control study, the association of venous thromboembolism and levels of IL, IL-6, IL-8, and monocyte chemotactic protein-1 (MCP-1) was examined.^13^ Blood was collected > 7 months after the thrombotic episode. Odds ratios (OR) adjusted for age and sex were 1.520 for IL-6, 1.1 for IL-8, and 1.0 for MCP-1. Polymorphisms did not affect the thrombotic risk and the cytokine levels in study participants.

In another study, it was demonstrated that patients with idiopathic venous thrombosis had significantly lower levels of IL-10. Patients also had increased levels of proinflammatory cytokines such as IL-6 and IL-8.^14^ In patients with trauma, an elevated serum P-selectin to IL-10 ratio was associated with the development of venous thromboembolism.^15^

It is noticeable that in our study the level of IL-6 was lower in the CVST patients in comparison with the control group that may stem from the chronic consumption of warfarin by the patients. According to some previous studies, warfarin may directly influence inflammatory signal transduction, in lower concentrations lessening such signaling but in higher concentrations provoking proinflammatory reactions; in other words, low-dose warfarin may have anti-inflammatory effect through suppression of IL-6 secretion and obstructing the immune-associated signals.^16^^,^^17^

The most important limitation of our study was the small sample size that is, as a result of scarcity CVST in comparison with other forms of venous thrombosis such as DVT in the extremities. Conducting study as multi-center study may solve this problem.

For future, we propose the measurements of ILs in a larger population; what is more, we suggest the measurements in acute phase as well as measuring more widespread vascular factors such as vascular cell adhesion molecule 1, intercellular adhesion molecule 1, and E-selectin and other related factors.

## Conclusion

According to our results, we did not find higher concentrations of pro-inflammatory cytokines in the patients with the history of CVST that is contradictory with some findings in venous thrombosis of the extremities; however, the studies with larger sample size may be required.

## Notes:

**How to cite this article:** Akbari F, Ghorbani A, Fatehi F. The assessment of proinflammatory cytokines in the patients with the history of cerebral venous sinus thrombosis. Iran J Neurol 2016; 15(2): 75-9.
